# Mammographic breast density and the risk of breast cancer: A systematic review and meta-analysis

**DOI:** 10.1016/j.breast.2022.09.007

**Published:** 2022-09-26

**Authors:** F.T.H. Bodewes, A.A. van Asselt, M.D. Dorrius, M.J.W. Greuter, G.H. de Bock

**Affiliations:** a Department of Epidemiology, University Medical Center Groningen (UMCG), University of Groningen, Hanzeplein 1, HPC: FA40, PO Box 30.001, Groningen, 9700 RB, the Netherlands; b Department of Radiology, University Medical Center Groningen (UMCG), University of Groningen, Groningen, the Netherlands

**Keywords:** Breast cancer risk, Breast cancer, Breast density, Mammographic density, BI-RADS

## Abstract

**Objectives:**

Mammographic density is a well-defined risk factor for breast cancer and having extremely dense breast tissue is associated with a one-to six-fold increased risk of breast cancer. However, it is questioned whether this increased risk estimate is applicable to current breast density classification methods. Therefore, the aim of this study was to further investigate and clarify the association between mammographic density and breast cancer risk based on current literature.

**Methods:**

Medline, Embase and Web of Science were systematically searched for articles published since 2013, that used BI-RADS lexicon 5th edition and incorporated data on digital mammography. Crude and maximally confounder-adjusted data were pooled in odds ratios (ORs) using random-effects models. Heterogeneity regarding breast cancer risks were investigated using I^2^ statistic, stratified and sensitivity analyses.

**Results:**

Nine observational studies were included. Having extremely dense breast tissue (BI-RADS density D) resulted in a 2.11-fold (95% CI 1.84–2.42) increased breast cancer risk compared to having scattered dense breast tissue (BI-RADS density B). Sensitivity analysis showed that when only using data that had adjusted for age and BMI, the breast cancer risk was 1.83-fold (95% CI 1.52–2.21) increased. Both results were statistically significant and homogenous.

**Conclusions:**

Mammographic breast density BI-RADS D is associated with an approximately two-fold increased risk of breast cancer compared to having BI-RADS density B in general population women. This is a novel and lower risk estimate compared to previously reported and might be explained due to the use of digital mammography and BI-RADS lexicon 5th edition.

## Highlights

• So far, breast density was linked to a one-to sixfold increased breast cancer risk.
• Prior risk estimates were based on old BI-RADS lexicon and screen-film mammography.
• We included only studies using digital mammography and BI-RADS lexicon 5th edition.
• Breast density D is associated with a two-fold increased risk of breast cancer.
• This is a lower risk estimate compared to previously reported studies.

## 1 Introduction

Breast cancer is the most common cancer and the leading cause of cancer-related death among women in Western countries [1]. Early detection of breast cancer, which has shown to reduce breast cancer-related burden and mortality, is warranted [[2], [3], [4], [5]]. Therefore, several countries have implemented a nationwide breast cancer screening programme in which women are screened by mammography on a regular base. In most countries, women between the age of 50 and 69 are screened, as these women are considered as the most appropriate group to benefit from this screening [2,6]. Increasingly, the potential of personalised risk-based breast cancer screening is examined, wherein women are offered screening strategies in which the screening frequency and modality are based on their risk to develop breast cancer [7]. Women that might benefit from such a personalised screening programme are those with mammographic dense breast tissue, and in 2022 the European Society of Breast Imaging (EUSOBI) announced to now recommend offering screening breast MRI every 2–4 years in women aged 50–70 years with extremely dense breasts [[7], [8], [9]].

Dense breast tissue refers to the amount of radiologically dense, fibro-glandular tissue in the breast [10,11]. Dense breast tissue is linked to markedly reduced mammographic sensitivity and a higher interval cancer rate [[11], [12], [13], [14], [15], [16]]. In addition, having dense breast tissue is suggested to be related to an increased risk of breast cancer compared to women with fatty breast tissue [11,17]. However, there is a high variability in the reported degree of increased breast cancer risk, ranging between a one-to sixfold increased risk [11,17]. The main explanation might be the use of various breast density indices in these studies, such as BI-RADS lexicon, Wolfe, Tabár and automated quantitative density measures [18,19]. Furthermore, the relationship of density with risk has predominantly been established using film-screen mammograms, which has been largely replaced by digital mammography [[20], [21], [22]]. Nowadays, the most commonly used tool for classifying mammographic density clinically is the Breast Imaging Reporting and Data Systems 5th edition (BI-RADS), and was introduced in 2013. This system defines four categories of breast density from extremely fatty (A), scattered density (B), to heterogeneous density (C), and extremely dense (D), of which category B is most prevalent among women of breast cancer screening age [17,23]. Yet, few studies have used BI-RADS 5th edition breast density to predict breast cancer risk and few have analysed the breast cancer risk in comparison to women with average dense breast tissue [17,22,24].

Therefore, the aim of this systematic review and meta-analysis was to further investigate the association between mammographic density and the risk of breast cancer, in terms of odds ratio, concerning women with extremely dense breast tissue (BI-RADS D) compared to women with average dense breast tissue (BI-RADS B), when only data on digital mammography and BI-RADS lexicon 5th edition are included.

## 2 Methods

This systematic review and meta-analysis is registered in PROSPERO (registration number CRD42022309522, https://www.crd.york.ac.uk/PROSPERO/).

A systematic review and meta-analysis was performed by two independent reviewers (FTHB and AAvA) according to a predetermined protocol based on the PRISMA guidelines [25]. Disagreements between the two reviewers were resolved by consensus and if consensus was not reached, a third reviewer (GHdB) was consulted. Studies were searched that included women of 18 years and older who underwent breast imaging, were classified as having dense breasts on mammography, and were monitored on the development of breast cancer.

### 2.1 Data sources and searches

A systematic search of PubMed, Embase and Web of Science was performed (last search date February 14, 2022). Relevant English-language articles published between January 2013 (BI-RADS 5th edition [23]) up to and including February 2022 were searched. Additionally, the bibliography of identified relevant articles and reviews were manually screened for additional eligible studies. The search strategy included three search strings: breast cancer, breast density, and risk in title or abstract (Appendix A).

### 2.2 Eligibility criteria

Eligible studies were: studies of an observational design that described the relationship between breast density and breast cancer risk; compared breast cancer risk to that in women with non-dense breasts; included at least 25 women with dense breasts; and where ‘dense’ breast tissue was defined as breast composition C and D (5th edition) or quantitative measurements with the same categories, e.g. Volpara or Quantra [23,26]. The fourth edition BI-RADS lexicon has been replaced since 2013 by BI-RADS lexicon fifth edition. In the fifth edition, percentage quartiles are removed and class descriptors were adjusted with the goal to better identify women whose cancers may be masked by dense breast tissue [23]. Articles had to be available in full-text, peer-reviewed, written in English and had to contain original data. If multiple articles were based on the same study population, the most extensive study (in terms of reported data) was chosen (Appendix B).

### 2.3 Study selection

Identified articles were de-duplicated using Endnote and afterwards loaded into Rayyan [27,28]. Titles and abstracts, followed by full-text, were screened based on the eligibility criteria and relevant articles were selected.

### 2.4 Data collection process

A data extraction sheet was developed and used to extract information from included studies: bibliographic information, type of study, study setting (blinding, study selection), number of women with dense breasts and non-dense breasts, inclusion and exclusion criteria, age of women (age of whole study population if not specified), length of follow-up, breast density index, number of breast cancers, reading protocol, definition for breast cancer (in- or excluding ductal carcinoma in situ (DCIS)), reported outcomes of breast cancer risk (relative risk (RR), hazard ratio (HR) or odds ratio (OR), adjusted and unadjusted), adjustments made for potential confounders, and their 95% confidence interval (95%CI) (Appendix C). In case multiple breast density indices were reported, the qualitative measure was used. For studies that reported their results in terms of RR or HR, the unadjusted crude data of affected and unaffected women were extracted as well.

### 2.5 Methodological quality and risk of bias assessment

The methodological quality of the included studies was assessed using the Newcastle-Ottawa Scale (NOS), which was adjusted to the requirements of this study, see appendix D [29]. The domains considered were: selection, comparability and assessments of outcome for cohort studies; and selection, comparability and ascertainment of exposure for case-control studies. The assessment was performed by two reviewers independently and final quality assessment was based on consensus. Methodological shortcomings were defined as >50% of the studies did not score a star on this item. The inter-rater agreement was evaluated by calculating percentage agreement between the reviewers and Cohen's kappa.

### 2.6 Statistical analysis

To investigate the association between breast density and breast cancer risk in women with dense breast tissue (BI-RADS C, and D) compared to women with average breast density (BI-RADS B), data were pooled under the assumption of homogeneity using a random-effects model [30]. In this way, summary odds ratios (OR) and related 95% confidence intervals were obtained. Unadjusted as well as maximally-adjusted risk estimates were analysed separately. For studies that reported their results in terms of RR or HR, calculation of the crude ORs, and related 95% CIs was based on the unadjusted crude data [30]. Results were presented in forest plots.

I^2^ statistic was used to evaluate heterogeneity [31]. Heterogeneity was assumed to be present if the I^2^ was >50% or when the chi-square was statistically significant (P < 0.05) [32].

Potential explanations for heterogeneity of breast cancer risks were explored (if there was any) by inspecting forests plots, and by stratified analyses and sensitivity analyses. Stratified analyses were performed to investigate the effect of covariates (e.g. age and BMI), outcome definitions (e.g. inclusion of DCIS), breast density indices (qualitative and quantitative measures), and study design (case-control and cohort studies). Stratified analyses were performed only when two or more studies were found in the subgroups. Sensitivity analyses were performed in which only maximally-adjusted risk estimates that had adjusted for age and at least one other covariate (e.g. BMI) were included. A significance level of P < 0.05 was used and all tests were two-sided.

To evaluate publication bias, a funnel plot of the log odds ratio against its standard error was produced to visually assess funnel plot asymmetry (an indication of the presence of publication bias) [33]. A significance level of P < 0.10 was used. Review Manager was used to enter and analyse all data [34].

## 3 Results

### 3.1 Retrieved studies

**Fig. 1:**
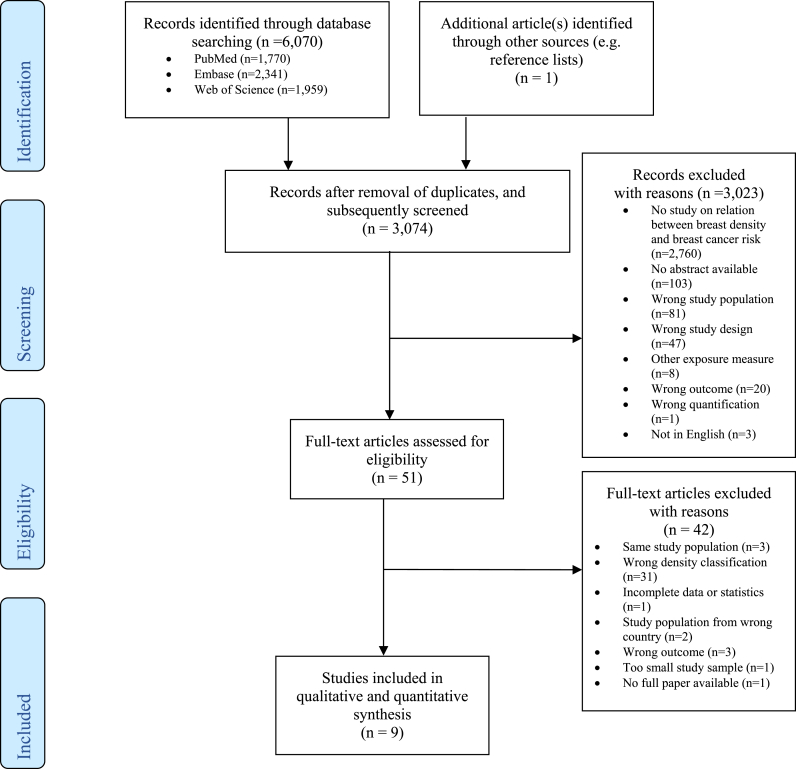
Flow diagram.

The search yielded 6071 articles (Fig. 1). One additional article was acquired by checking the references of relevant articles. After removal of 2997 duplicates, 3074 studies were screened on title and abstract and 3023 were eliminated as these papers did not meet the inclusion criteria. This led to 51 articles of which the full-text was examined in more detail. Finally, 42 articles were excluded, for reasons see Fig. 1, leaving nine articles to be included in this systematic review and meta-analysis [[35], [36], [37], [38], [39], [40], [41], [42], [43]].

### 3.2 Study characteristics

All nine included studies investigated in total 386,590 women on mammographic density and breast cancer risk, with 6 case-control studies [[35], [36], [37], [38], [39], [40]], and 3 cohort studies [[41], [42], [43]]. The studies represented data for a total of 11,253 cases and 375,337 non-cases of breast cancer. Included were women who participated in a population screening program [[35], [36], [37],[39], [40], [41], [42], [43]] or in a study cohort [38]. All studies investigated predominantly women between 50 and 70 years, and seven studies reported a median or mean women's age of early to late fifty [35,36,38,[40], [41], [42], [43]]. All studies were conducted in Western or developed countries.

All studies used either record linkage or medical records to objectively determine the presence of breast cancer [[35], [36], [37], [38], [39], [40], [41], [42], [43]]. Seven studies defined breast cancer as the presence of invasive cancer or DCIS [[35], [36], [37], [38],[41], [42], [43]], and two studies included solely data on invasive breast cancer [39,40]. Study subjects with prevalent breast cancers or breast cancers that were diagnosed in the first screening round were excluded in six studies [[35], [36], [37],[41], [42], [43]]. All studies defined controls or non-cases as having no diagnosis of breast cancer [[35], [36], [37], [38], [39], [40], [41], [42], [43]]. Four studies objectively examined breast density by using the automated volumetric method Volpara [35,36,41,42]. Four studies assessed breast density subjectively using BI-RADS (5th edition) [37,38,40,43]. One study examined breast density by both methods [39].

With regard to the risk estimates, all studies reported crude, numerical data. Additionally, six studies reported adjusted ORs [35,36,[38], [39], [40],^43^], one study reported cases/person-years RR [41], and one study reported adjusted HR [42]. All studies compared breast cancer risk of women with dense breasts to lower density categories (BI-RADS C or D vs. A) [[35], [36], [37], [38], [39], [40], [41], [42], [43]]. Five studies reported adjusted risk estimates with BI-RADS B as reference group [39,40,[42], [43], [44]]. Of the six studies that reported adjusted risk estimates in terms of odds ratios [35,36,[38], [39], [40],^43^], one study only adjusted for age [43] while the other five studies adjusted for two or more covariates [38,39,[41], [42], [43]]. (Appendices E and F).

### 3.3 Methodological quality

The methodological quality as assessed by the scale varied from five to seven stars for case-control studies [[35], [36], [37], [38], [39], [40]] and for cohort studies [[41], [42], [43]]. Most methodological shortcomings according to the NOS in case-control studies were with regard to [1]: inadequacy of case definition (often obtained through record linkage, 83.3% of the studies); and [2] non-response rates for cases and controls (83.3% of the studies). For cohort studies, most methodological shortcomings according to the NOS were with regard to [1]: unclear that the outcome of interest was not present at the start of the study (66.7% of studies); and [2] (description of) adequacy of follow-up cohorts (66.6%). The inter-rater agreement on the NOS items was high (overall agreement 97.5% (79/81), Cohen's kappa 0.943, p < 0.0001). (appendix G, table G.1).

### 3.4 Results of meta-analysis

Tables F1 and F.2 in appendix F provide an overview of the reported risk estimates presented per study. All studies reported an increased risk of breast cancer in women with BI-RADS D compared to women with BI-RADS A. For the meta-analysis, results of two studies could not be included in the maximally confounder-adjusted meta-analysis due to insufficient reported data [37,41].

For crude data, a pooled OR of 1.63 (95% CI = 1.36–1.95; I^2^ = 82%, p < 0.00001; figure H.1) was found for women with BI-RADS D compared to women with BI-RADS B, and a pooled OR of 2.33 (95% CI = 1.95–2.78; I^2^ = 72%, p < 0.00001; figure H.3) for women with BI-RADS D compared to women with BI-RADS A as defined in the included studies. When comparing the breast cancer risk of women with BI-RADS C to women with BI-RADS B the pooled OR was 1.28 (95% CI = 1.19–1.37; I^2^ = 37%, p < 0.00001; figure H.5).

**Fig. 2:**
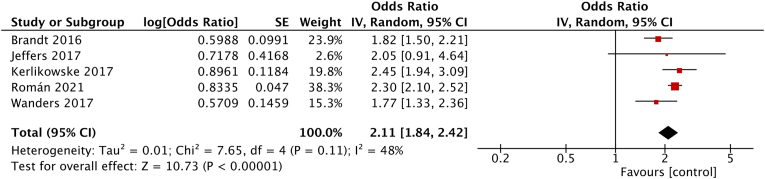
Forest plot of the pooled risk (95% CI) for five included studies. Breast cancer risk for women with BI-RADS density D compared to women with BI-RADS B. Maximally-adjusted data were used. Standard Error (SE); Confidence Interval (CI).

Five studies reported adjusted risk estimates with the BI-RADS B as reference group. The pooled estimate of the maximally confounder-adjusted data reported by these studies showed a pooled OR of 2.11 (95% CI = 1.84–2.42; I^2^ = 48%, p < 0.00001; Fig. 2) among women with BI-RADS D in comparison to women with BI-RADS B, and a pooled OR of 3.89 (95% CI = 2.47–6.13; I^2^ = 58%, p < 0.00001; figure H.8) for women with BI-RADS D compared to women with BI-RADS A.

The results were heterogeneous according to the aforementioned heterogeneity criteria except for the pooled estimate of the maximally confounder-adjusted data comparing BI-RADS D to BI-RADS A, and breast cancer risks were higher when the maximally-adjusted data were pooled. All forests plots and its corresponding funnel plots are depicted in appendix H.

Stratified analyses of the maximally-adjusted data that used BI-RADS density B as reference group, were performed to explore heterogeneity and breast cancer risks. It was found that breast cancer risks were higher than the pooled OR of 2.11 (95% CI 1.84–2.42) for studies that used qualitative BI-RADS to categorize breast density and for studies that included invasive breast cancer cases only. All stratified analyses are presented in Table 1.

**Table 1:** Stratified analyses for maximally-adjusted data.

Stratification by	Studies	Pooled OR (95% CI), p-value	I^2^ (%)	P-value heterogeneity
**Type of breast density index**				
VDG^a^	3	1.81 (1.55–2.12)**	0	0.04
BI-RADS Density^b^	2	2.32 (2.13–2.53)**	0	0.62
**Type of outcome measurement**^a^				
Invasive and DCIS^c^	3	2.00 (1.66–2.43)**	70	0.04
Invasive only	2	2.42 (1.93–3.02)**	0	0.68
**Study design**				
Case-control	3	2.09 (1.65–2.63)**	46	0.16
Cohort	2	2.09 (1.63–2.68)**	66	0.09
**Studies adjusted for:**				
**Age and BMI**^d^				
Yes	2	1.83 (1.52–2.21)**	0	0.78
No	2	2.09 (1.63–2.68)**	66	0.09

*p-value for overall effect was <0.01.

**p-value for overall effect was <0.00001.

a Volpara Density Grade (VDG).

b Breast-Imaging Reporting and Data System (BI-RADS).

c Ductal Carcinoma in Situ (DCIS).

d Body Mass Index (BMI).

In the sensitivity analysis, the pooled OR showed an increased risk of 1.83 (95% CI 1.52–2.21; I^2^ = 0%, p < 0.00001) for the two studies that had adjusted for age and BMI (figure H.13).

### 3.5 Publication bias and funnel plot (a)symmetry

**Fig. 3:**
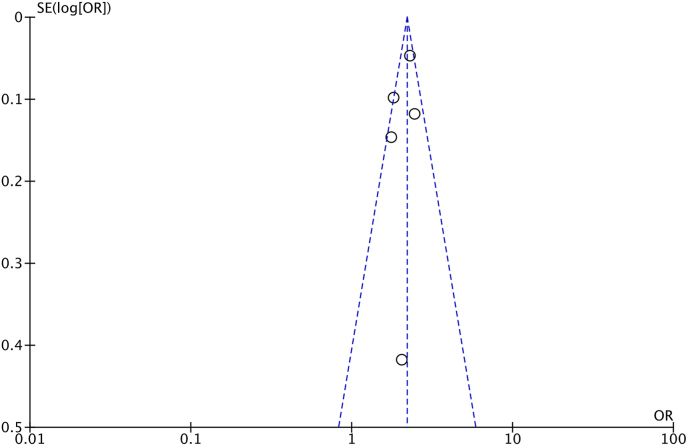
Funnel plot of the breast cancer risk in five included studies in which effects estimates (ORs) are plotted against their standard errors (SEs) in a fixed-effect meta-analyses. Maximally-adjusted data were used.

Fig. 3 depicts the funnel plot of the nine included studies. Visual inspection of the funnel plot indicated that publication bias was unlikely to be present. Fewer than 10 studies were included, so funnel plot asymmetry was not statistically tested [45].

## 4 Discussion

This systematic review and meta-analysis investigated studies published from 2013 that included data on digital mammography, assessing the association between mammographic density and breast cancer risk from nine observational studies among general population women [[35], [36], [37], [38], [39], [40], [41], [42], [43]]. In our study, only the most recent and widely used BI-RADS 5th edition density measures and automated density measuring methods using the same categories were included. Having BI-RADS density D resulted in a 2.11-fold (95% CI 1.84–2.42) increased breast cancer risk compared to having BI-RADS B, and a 3.89-fold (95% CI 2.47–6.13) increased breast cancer risk compared to having BI-RADS density A. The sensitivity analysis showed that when adjusting for age and at least one other covariate (e.g. BMI) the breast cancer risk for women with BI-RADS density D in comparison to women with BI-RADS density B was 1.83-fold (95% CI 1.52–2.21) increased. All results were statistically significant.

Uniquely, we have provided a pooled OR of 1.83 (95% CI 1.52–2.21) comparing breast cancer risk in women with BI-RADS density D to BI-RADS density B, of which the latter is the most prevalent category in women of breast cancer screening age [43]. Additionally, we have provided a risk estimate comparing breast cancer risk in women with BI-RADS density C to women with BI-RADS density B, showing an 1.28-fold (95% CI 1.19–1.37) increased breast cancer risk. The increased breast cancer risk (OR 3.89) found among general population women with BI-RADS density D compared to women with BI-RADS density A is lower than what has been reported by previous reviews as by Boyd et al. [24] and McCormack et al. [17]. In these studies higher risk estimates ranging from a four-to five-fold increased risk in women with >75% density compared to women with little or no density are reported.

Our lower risk estimate could be due to various aspects, such as the use of newer methods of mammography (namely digital mammography), more widely used and different density classifications (namely BI-RADS lexicon 5th edition), and particularly a difference in reference groups (namely BI-RADS B, instead of BI-RADS A or women with little or no density). Our lower risk estimate might therefore be a more accurate reflection of the current breast cancer risk.

### 4.1 Current density measurement

Digital mammography was developed in part to improve the detection of breast cancer in women with dense breasts. In many Western countries, digital mammography has replaced screen-film mammography. Digital mammography is associated with higher accuracy and higher detection rates of breast cancer compared to screen-film mammography, also for women with dense breasts [[46], [47], [48]]. As digital mammography has improved contrast resolution compared to screen-film mammography, breast density is lower for digital than for screen-film mammography, though this was never investigated for BI-RADS lexicon fifth edition [49,50]. The few studies that have studied the association of mammographic density with breast cancer risk using digital mammography also show that the association is slightly weaker when breast density is assessed with digital mammography compared to screen-film mammography [36,39,51,52]. This is in line with our lower risk estimate compared to previous studies.

The included studies provided data on quantitatively and qualitatively measured BI-RADS lexicon 5th edition. Previously, there was no consensus on which density measurement most accurately reflects true breast density and there was a historic lack of standardized breast density classification criteria [18,19]. However, BI-RADS 5th edition is currently the most widely used category for classifying mammographic density and radiologists routinely report the breast density when assessing a mammogram [17,23,36,43]. In the fifth edition, percentage quartiles are removed with the goal to better identify women whose cancers may be masked by dense breast tissue [23]. Multiple studies have shown that BI-RADS lexicon 5th edition is associated with significant higher numbers of dense assessments compared to the fourth edition [53,54]. This might also explain the lower risk estimate than previously reported.

### 4.2 Automated density assessment methods

With the introduction of digital mammography, automated density assessment methods are being researched and developed [35]. Some automatic volumetric density assessment methods have classification systems corresponding to the BI-RADS density categories [36,[55], [56], [57]]. Objective measurement tools could be useful in improving the reproducibility of results, or when programs do not routinely determine breast composition [43]. Qualitative density, such as BI-RADS lexicon, measures are categorized as subjective and have been linked to inter-reader variability and misclassification [58]. Several studies are being conducted in which qualitative and quantitative density assessment methods are being compared. In these studies, all density measures were positively associated with breast cancer risk, and in most studies, the clinical assessment with breast density categories allowed the best discrimination of patients from control subjects [36,39]. Our stratified analysis, as described in the results section, showed that quantitative measurements were associated with lower breast cancer risk than qualitative measurements, which is in line with the study of Brandt et al. (2015) [36]. This could be explained since qualitative and quantitative breast density measures both measure different aspects of breast density: BI-RADS density categories are assessed visually and reflect density quantity, distribution, and parenchymal pattern, while quantitative measures algorithmically assess absolute dense volume or area [59]. Future studies are being performed in order to investigate the breast cancer risk according to automated density assessment methods.

### 4.3 Confounders

Adjusting for confounders increased the breast cancer risk in our analysis from OR 1.63 (95% CI = 1.36–1.95) to OR 2.11 (95% CI = 1.84–2.42) when comparing BI-RADS density D to BI-RADS density B. Breast density can be affected and confounded by age, obesity, obstetrical history, menopausal status, HRT, age, family history, and genotype [[60], [61], [62]]. Previous studies have shown that failure to adjust for BMI could lead to an underestimation of the association [17,63]. On the contrary, increasing age is associated with a decline in breast density [64].

Factors such as a strong family history for breast cancer, nulliparity, premenopausal status, and HRT use increase mammographic density, and higher oestrogen levels have been associated with increased breast cancer risk [[65], [66], [67], [68]]. Taking into account and adjusting for these confounding factors may thus influence the risk estimates and need to be considered when conducting further research.

### 4.4 Limitations and strengths

This systematic review has a few potential limitations. Firstly, methodological variety existed between studies, and statistical heterogeneity was present in several analyses. Exploring heterogeneity with stratified and sensitivity analyses explained reasons of heterogeneity and showed homogenous risk estimates, namely when stratification occurred by type of breast density index, outcome, and adjusting for age and BMI. Secondly, there was variation with regard to the outcome of breast cancer, and more specifically with regard to the in- or exclusion of DCIS in breast cancer cases. Additionally, there was some but no complete overlap, between the study subjects of the two articles, namely the study by Brandt et al. and Kerlikowske et al. [36,40]. This was incorporated in the sensitivity analysis, in which data from Kerlikowske et al. was excluded [40]. In many studies, covariates were at least partly based on self-reports or questionnaires, which could have resulted in misreporting and recall bias. The relatively small number of studies included is a shortcoming, but data on a large number of women were analysed. Further limitations entail density misclassification and inherent potential biases; the retrospective design of some included studies; and the possibility of incomplete retrieval of relevant studies. Strengths include that the methodological quality of included studies was moderate (all studies obtained five to seven stars), and the presence of publication bias was found to be unlikely. Supplemental strengths of this study were the large predominantly Caucasian population investigated, only countries were included in which all populations were at generally high risk of breast cancer [69], and the identification of gaps in literature, such as the limited number of studies assessing breast density and breast cancer risk according to BI-RADS 5th edition, and comparing risk to women with average breast density.

### 4.5 Implications

Breast density is a risk factor for breast cancer that is easy to measure, might be modifiable and influenced by other factors, and overall knowledge on breast density may influence clinical management. Our study showed that breast density is related to a two-fold increased breast cancer risk. It can be questioned whether a two-fold risk is strong enough to advocate breast MRI for women with dense breasts. This risk estimate is equal to the lowest risk estimate associated with a strong family history of breast cancer, which is associated with a two-to three-fold increased breast cancer risk, and is therefore considered as substantial, also given the high lifetime risk for breast cancer in general population women of about 15% [68,70]. Combined with the reduced sensitivity of mammography in women with dense breasts, we strongly advise to screen women with dense breast tissue with MRI [13]. This is in line with a recent recommendation from the EUSOBI which now recommends that women should be informed about their breast density and recommends screening breast MRI to women with extremely dense breasts [9].

### 4.6 Conclusion

This systematic review, based on data of 386,590 women, provides further evidence that having extremely dense breast tissue (BI-RADS density D) is significantly associated with increased breast cancer risk compared to having scattered dense breast tissue (BI-RADS density B), when including only density measures that are nowadays clinically widely used. Our risk estimate is lower than was previously established and is a more accurate reflection of the current breast cancer risk. Nonetheless, the findings from this systematic review still support and highlight the potential of supplemental MRI screening and other intervention methods for women with dense breast tissue to earlier detect and reduce breast cancer cases.

## Funding source

This research did not receive any specific grant from funding agencies in the public, commercial, or not-for-profit sectors.

## Declaration of competing interest

The authors declare that they have no known competing financial interests or personal relationships that could have appeared to influence the work reported in this paper.
