# Supplementary material for: Mammographic breast density and the risk of breast cancer: A systematic review and meta-analysis
Source: Breast. 2022 Sep 26;66:62–8. doi: 10.1016/j.breast.2022.09.007 (PMC9530665; doi:10.1016/j.breast.2022.09.007)
Supplement: Multimedia component 1 [file mmc1.docx]

# **Appendices**

**Appendix A – Search Strategy**

**PubMed**

(“Breast neoplasms”[Mesh] OR breast neoplasm*[tiab] OR breast cancer*[tiab] OR breast tumo*[tiab] OR breast carcinoma*[tiab])

AND

(“Breast density”[Mesh] OR dense breast*[tiab] OR breast dens*[tiab] OR mammographic dens*[tiab] OR mammographically dens*[tiab] OR tissue dens*[tiab] OR dense tissue[tiab] OR mammographic parenchym*[tiab] OR breast pattern*[tiab] OR mammographic pattern*[tiab] OR parenchymal pattern*[tiab])

AND

("Cohort Studies"[Mesh] OR "Case-Control Studies"[Mesh] OR "Cross-Sectional Studies"[Mesh] OR "Risk Assessment"[Mesh:NoExp] OR "Risk Factors"[Mesh] OR cohort*[tiab] OR case-control[tiab] OR associat*[tiab] OR risk factor*[tiab] OR risk[ti] OR relat*[ti] OR predict*[ti])
AND
("2013/01/01"[pdat] : "3000"[pdat])

**Embase**

('breast tumor'/exp OR (‘breast neoplasm*’ OR ‘breast cancer*’ OR ‘breast tumo*’ OR ‘breast carcinoma’):ti,ab)

AND
('breast density'/exp OR ((breast* OR mamma* OR mammo* OR tissue) NEAR/3 dens*):ab,ti OR (‘mammographic parenchym*’ OR ‘breast pattern*’ OR ‘mammographic pattern*’ OR ‘parenchymal pattern*’):ti,ab)

AND

('longitudinal study'/exp OR 'prospective study'/exp OR 'retrospective study'/exp OR 'cohort analysis'/exp OR 'case control study'/exp OR 'cross-sectional study'/exp OR 'risk assessment'/exp OR 'risk factor'/exp OR (cohort* OR ‘case-control*’ OR associat* OR ‘risk factor*’):ti,ab OR (risk OR relat* OR predict*):ti)
NOT
('conference abstract'/it OR ('animal'/exp NOT 'human'/exp))
AND
[2013-2021]/py

**Web of Science (Web of Science Core collection)**

TS= ((“breast neoplasm*“ OR “breast cancer*“ OR “breast tumo*“ OR “breast carcinoma*“)

AND

(“dense breast*“ OR “breast dens*“ OR “mammographic dens*“ OR “mammographically dens*“ OR “tissue dens*“ OR “dense tissue“ OR “mammographic parenchym*“ OR “breast pattern*“ OR “mammographic pattern*“ OR “parenchymal pattern*“)

AND

(longitudinal*[tiab] OR “follow up” OR prospectiv* OR retrospectiv* OR cohort* OR “case-control“ OR “cross-sectional*“ OR associat* OR risk OR relat* OR predict*))
AND
DT=(article)

# **Appendix B - Eligibility criteria**

Risk of breast cancer in women with dense breast tissue

1. Research must be:

- 1. In English YES / NO
  2. A full-text publication YES / NO
  3. Published after 2013 YES / NO

2. The study must describe the relationship between breast density YES / NO

and breast cancer risk.

Breast cancer was defined as a biopsy-confirmed breast carcinoma.

3. Quantification of the breast cancer risk in relation to breast density YES / NO

was given in terms of relative risk, hazard ratio or odds ratio.

4. Study must be an observational study: a case-control study YES / NO

or cohort study, including longitudinal, follow-up, retrospective

and prospective studies.

Studies of other designs, such as randomized controlled trials, and other publication types (e.g. meta-analyses, reviews or editorials) are excluded.

5. Study population consists of women, with at least 20 women per category. YES / NO

The study should involve adult women (>18 years) of all ages. The study population should mostly contain women from a Caucasian descent. Study populations from an Asian, Middle-East and South-American descent (e.g. China, India, Iran) will be excluded. Genetic predisposition such as BRCA1^1^ or BRCA2 should not be present in these women.

6. Breast density should be assessed and defined according to YES / NO

BI-RADS^2^ 5^th^ edition, Volpara or quantitative measurements with the same categories and should be categorized in category c, d and compared with women with category a and/or b or 0-50%.

Density of the breast tissue is determined either prior or after the diagnosis of breast cancer.

**Study is included / excluded / other (circle)**

1 Breast Cancer Gene (BRCA); 2 Breast-Imaging Reporting and Data System (BI-RADS)

# **Appendix C - Data extraction sheet**

- Bibliographic information
  - Author(s)
  - Year of publication
  - Journal
- Study design and study characteristics;
  - Design: A case-control study or cohort study, including longitudinal, follow-up, retrospective and prospective studies
  - Blinding of density assessors to cancer status
  - Use of concurrent or prospective mammography
  - Population selection: screening or diagnostic
  - Study period
- Study population:
  - Cases
    - Nationality
    - Ethnicity
    - Characteristics
      - Age (range), mean
      - Other known variables/characteristics, e.g. Body Mass Index (BMI), menopausal status, parity
    - Inclusion criteria
    - Exclusion criteria
    - Information obtained from, e.g. medical record, questionnaire, interview
    - Sample size
  - Controls
    - Characteristics
    - Matching variables if appropriate
    - Sample size
- Determinant: mammographic density
  - Type of breast density measurement index (Breast-Imaging Reporting and Data System (BI-RADS), Volpara etc.)
  - Mammogram view
  - Type of image, e.g. digital or analog
  - Assessment by: (number of) radiologist(s), experienced or not
- Outcome: breast cancer cases
  - Breast cancer definition, e.g. as biopsy-confirmed breast carcinoma, in- or exclusion of ductal carcinoma in situ (DCIS) cases.
  - Inclusion of both interval and screen-detected breast cancer cases
- Results
  - Numerical results: number of participants per group, number with outcome
  - Risk estimates for breast cancer associated with breast density and their 95% Confidence Intervals (CIs).
    - Adjusted and unadjusted Relative Risk (RR), Odds ratio (OR) or Hazard Ratio (HR) and their 95% CI and p-values.
      - Report adjustments made
- Other:
  - Comments on particularities

# **Appendix D - Newcastle-Ottawa Scale (NOS) quality assessment**

## **Case Control Studies**

Note: A study can be awarded a maximum of one star for each numbered item within the Selection and Exposure categories. A maximum of two stars can be given for Comparability.

*Selection*

1) Is the case definition adequate?

a) Yes, with independent validation (e.g. reference to primary record source such as medical record) **🟑**

b) Yes, e.g. record linkage (e.g. ICD codes in database) or based on self-reports with no reference to primary record

c) No description

2) Representativeness of the cases

a) Consecutive or obviously representative series of cases **🟑**

b) Potential for selection biases or not stated

3) Selection of Controls

a) Community controls **🟑**

b) Hospital controls

c) No description

4) Definition of Controls

a) No history of disease (Breast cancer as endpoint) **🟑**

b) No description of source/No mention of history of outcome

*Comparability*

1) Comparability of cases and controls on the basis of the design or analysis

a) Study controls for age **🟑**

b) Study controls for any additional factor, such as Body Mass Index (BMI), parity, menopausal status **🟑**

*Exposure*

1) Ascertainment of exposure

a) Secure record (e.g. surgical records) **🟑**

b) Structured interview/assessment of exposure where blind to case/control status **🟑**

c) Ascertainment of exposure not blinded to case/control status

d) Written self-report or medical record only

e) No description

2) Same method of ascertainment for cases and controls

a) Yes **🟑**

b) No

3) Non-Response rate

a) Same rate for both groups **🟑**

b) Non respondents described

c) Rate different and no designation
 d) Not stated

## **Cohort Studies**

Note: A study can be awarded a maximum of one star for each numbered item within the Selection and Outcome categories. A maximum of two stars can be given for Comparability

*Selection*

1) Representativeness of the exposed cohort

a) Truly representative of the average woman in the community **🟑**

b) Somewhat representative of the average woman in the community **🟑**

c) Selected group of users e,g. nurses, volunteers

d) No description of the derivation of the cohort

2) Selection of the non-exposed cohort

a) Drawn from the same community as the exposed cohort **🟑**

b) Drawn from a different source

c) No description of the derivation of the non-exposed cohort

3) Ascertainment of exposure

a) Secure record (e.g. surgical records) **🟑**

b) Structured interview/assessment of exposure **🟑**

c) Written self-report

d) No description

4) Demonstration that outcome of interest was not present at start of study

a) Yes **🟑**

b) No

*Comparability*

1) Comparability of cohorts on the basis of the design or analysis

a) study controls for age **🟑**

b) study controls for any additional factor, such as Body Mass Index (BMI), parity, menopausal status **🟑**

*Outcome*

1) Assessment of outcome

a) Independent blind assessment **🟑**

b) Record linkage **🟑**

c) Self-report

d) No description

2) Was follow-up long enough for outcomes to occur

a) Yes, follow-up was > 3 years **🟑**

b) No

3) Adequacy of follow up of cohorts

a) Complete follow up - all subjects accounted for **🟑**

b) Subjects lost to follow up unlikely to introduce bias - small number lost - > 80 % follow up, or description provided of those lost indicating no differences on e.g. age **🟑**

c) Follow up rate < 80% and no description of those lost

d) No statement

# **Appendix E - Evidence table**

Table E.1: Evidence table

| **Author and Publication Year** | **Astley SM (2018)** | **Brandt KR (2016)** | **Engmann NJ (2019)** | **Eriksson M (2017)** | **Jeffers AM (2017)** | **Kerlikowske K (2017)** | **Puliti D (2018)** | **Román M (2021)** | **Wanders JOP (2017)** |
| --- | --- | --- | --- | --- | --- | --- | --- | --- | --- |
| Journal | Breast Cancer Research | Radiology | Breast Cancer Research and Treatment | Breast Cancer Research | Radiology | Breast Cancer Research | Breast Cancer Research | European Radiology | Breast Cancer Research |
| **Study characteristics** |  |  |  |  |  |  |  |  |  |
| Design | CC^a^ | Retrospective CC | CC | CC | CC | CC | Cohort | Cohort | Cohort |
| Blinding | Yes | Not specified | No | Not specified | Yes | Not specified | Not specified | Not specified | Yes |
| Concurrent or prospective mammogram | Both | Prospective | Prospective | Prospective | Prospective | Prospective | Prospective | Prospective | Prospective |
| Population selection | Greater Manchester Breast Screening Service, PROCAS^b^ study | San Francisco Mammography Registry, Mayo Clinic | San Francisco Mammography Registry and Mayo Clinic, Rochester. | KARMA Cohort^c^ | Screening mammography at Stanford University | SFMR^d^, VBCSS^e^, Mayo Clinic Cohort | Florence screening programme | Spanish Breast Cancer Screening Program | Dutch Prevention screening unit In Utrecht |
| Study period | 2009-2015 | 2007-2012 | 2007-2015 | 2011-2015 | 2004-2013 | 2007-2013 | 2006-2013 | 1995-2017 | 2003-2011 |
| **Study population** |  |  |  |  |  |  |  |  |  |
| *Cases* |  |  |  |  |  |  |  |  |  |
| Nationality | British | American | American | Swedish | American | American | Italian | Spanish | Dutch |
| Ethnicity | >90% White | 73.4% Caucasian | >70% Caucasian | Not reported^f^ | Not reported^f,^ White, black, Asian, other | >80% White | Not reported | Not stated | Not stated |
| Characteristics  Age (range)  Other known variables, including confounder-adjusted factors | < 50 until 70+  Median: 58, 61, and 57 yrs  10-year Tyrer-Cuzick (v.6) risk score  Parity  Known: menopausal status; HRT^g^ use; BMI^h^; Ethnicity; Year of mammogram; family history; current alcohol use; any children; prior biopsy of breast | 49-68, median 58.0 yrs  Age; BMI^h^; race; number of years to diagnosis; invasive breast cancer; parous status; first-degree family history of breast cancer; postmenopausal HRT^g^ | <45 and >65 yrs, median premenopausal 45, post 63  Age; BMI;^h^ race; family history of breast cancer; age at first birth; menopausal status; current use of HRT^g^ | 40-79; Mean age at mammography 57.4  Age at breast cancer diagnosis, BMI^h^, age at menarche, parity, age at first birth, current use of HRT^g^, menopausal status, family history of breast cancer; microcalcifications; masses | <40 – 79 yrs  Age; menopausal status; parity; BMI^h^; race. | Mean (SD) 59.5 yrs (12.1)  Age; BMI^h^; age at first birth; family history of breast cancer; history of breast biopsy; race | 49-54 yrs  Median 50.9 yrs  Medical history of breast cancer; breast implants; screening histories | 50-69, >59% 50–54 yrs  Age at first screen, and year of screen, benign breast disease. | 50-75, median 56 yrs  - |
| Inclusion if | Women screened with mammography and breast cancer diagnosis  Women who were breast cancer free at the screen on entry to PROCAS^b^ but had breast cancer detected subsequently, either between screening rounds or at a later screen. | Women screened with mammography and incident breast cancer diagnosis, with availability of clinical risk factor data, BI-RADS^i^ density, raw FFDM^j^ images at least 6 months prior to receipt of the cancer diagnosis | Women with BC diagnosis and a screening FFDM^j^ at least 6 months prior to diagnosis. | Women who attended mammographic screening at four hospitals in Sweden were invited to be included | Women screened with mammography and subsequently breast cancer diagnosis | Breast cancer diagnosis after participation in screening program. | Women screened with mammography and subsequently breast cancer diagnosis | Women aged 50–69 years with at least one BI-RADS^i^ breast density examination at screening mammography between January 1996 and December 2015. | Women screened with mammography and subsequently breast cancer diagnosis |
| Exclusion: | If pre-existing diagnosis of breast cancer, synchronous bilateral breast cancer, film mammograms, moved out of the area. | If permission to use their medical records for research was refused | If data were missing on BMI^h^ or menopausal status. | If women were diagnosed with breast cancer within 3 months of a negative entry mammogram | Not specified. | Not specified | Previous breast cancer; breast implants; Previously enrolled in the active arm of an Italian study offering ultrasound in addition to screening mammography | Women diagnosed with breast cancer at first screen as they did not have any follow-up | All screen-detected breast cancers diagnosed as a result of first screening exam; Interval cancers diagnosed more than 24 months after a negative screening mammogram |
| Information obtained from | Mammogram, hospital records, North West Cancer Intelligence Service, self-reported questionnaire | California Cancer Registry, Mayo Clinic tumor registry, medical records | Self-reported via questionnaire, medical record, cancer registry | Web-based questionnaire, national breast cancer registers | Institutional cancer registry, medical records | Cancer registry, pathology databases, self-report, medical record | Tuscan Cancer Registry | Screening center databases, population-based cancer registries, the regional Minimum Data Set, and hospital-based cancer registries. | Screening registration system, the Netherlands Cancer Registry |
| *Controls* |  |  |  |  |  |  |  |  |  |
| Characteristics  Other variables same as cases | All had a subsequent cancer-free screening mammogram  Yes | Women without prior breast cancer | Women without breast cancer  Yes | Women included in cohort with no breast cancer. | Women without a history of breast cancer, at least 10 years of follow-up for women aged 50 years or older at mammography; three or more screening mammograms negative for cancer for women younger than 50 yrs at mammography.  Exclusion if history of breast cancer, breast cancer or breast implants in pathology reports | Women without prior breast cancer  Yes | NA^k^ | NA^k^ | Remaining cohort with no breast cancer  Yes |
| Matched variables | Age, BMI^h^ category, HRT^g^ use, menopausal status, Some controls were matched on year of mammogram at entry. | Age; race; FFDM^j^ examination date; imaging machine; facility; state of residence | Age, date of earliest mammogram, race/ethnicity, mammography facility, state of residence and mammography unit. | Age | 5-year age categories; race. | Age within 5 years, race, state of residence, date of screening, date of screening examination within 1 year, mammography machine, and facility | NA^k^ | NA^k^ | NA^k^ |
| *N (breast cancer cases: controls/ non-cases)* | VDG5^l^: 365:1,098  VDG5/2 study: 338:1,014 | 1,911:4,170 | 2,290:5,362 | 433:1,732 | 125:274 | 1,720;3,686 | 216 out of 15,952 | 3,697 out of 294,246 | 523 out of 52,291 |
| **Breast density** |  |  |  |  |  |  |  |  |  |
| Index | VDG^l^ 5^th^ edition | VDG^l^ and Quantra Density Assessment | BI-RADS^i^ 5^th^ edition | Stratus cBI-RADS^i^ categories | BI-RADS^i^ 5^th^ and VDG^l^ | BI-RADS^i^ 5^th^ edition | VDG^l^ | BI-RADS^i^ 5^th^ edition | VDG^l^ |
| View | Raw FFDM^j^ images for each view | Raw FFDM^j^ images for each view | MLO^m^ and CCv^n^ | MLO^m^ and CCv^n^ | CCv^n^ and raw (unprocessed) FFDM^j^ images | MLO^m^ and CCv^n^ | MLO^m^ and CCv^n^ | Not specified | MLO^m^ |
| **Author and Publication Year** |  |  |  |  |  |  |  |  |  |
| Type of image | FFDM^j^ | FFDM^j^ | FFDM^j^ | FFDM^j^ | FFDM^j^ | FFDM^j^ | FFDM^j^ | Film and digital mammography | FFDM^j^ |
| Radiologist(s) | Not applicable | Not applicable | Multiple | Not applicable | 1 | Multiple | Not applicable | Multiple | Not applicable |
| **Outcome** |  |  |  |  |  |  |  |  |  |
| Definition breast cancer | Invasive and DCIS^o^ | Invasive and DCIS^o^ | Invasive and DCIS^o^ | Invasive and DCIS^o^ | Invasive | Invasive | Invasive and DCIS^o^ | Invasive and DCIS^o^ | Invasive and DCIS^o^ |
| **NOS score (0-9)** | 7 | 7 | 5 | 6 | 5 | 6 | 5 | 7 | 6 |
| **Other** |  |  |  |  |  |  |  |  |  |
| Particularities | Divided into three study groups.  Also other density methods assessed (did not meet inclusion criteria). | Also other density methods assessed (BI-RADS^i^ 4^th^ edition) | Also analysis on influence of BMI^h^ performed. | - | Cumulus percentage of density quartiles also reported (data not shown). | - | Also data on interval and advanced cancers available. | Also, data available on invasive cancers only. | No adjustments other than for age. Also, data available on invasive cancers only. |

a. Case-control study (CC); b. Predicting the Risk of Cancer At Screening (PROCAS study); c. Karolinska Mammography Project for Risk Prediction of Breast Cancer (KARMA cohort); d. San Francisco Mammography Registry (SFMR) e. Vermont Breast Cancer Surveillance System (VBCSS); f. However, in all countries the majority of the population is of Caucasian origin; g. Hormone replacement therapy (HRT); h. Body Mass Index (BMI); i. Breast-Imaging Reporting and Data System (BI-RADS); j. Full-Field Digital Mammography (FFDM); k. Not Applicable (NA); l. Volpara Density Grade (VDG); m. Mediolateral oblique view (MLO); n. Craniocaudal view (CCv); o. Ductal Carcinoma in Situ (DCIS).

# **Appendix F – Summary of data**

Table F.1: Summary of the data from included articles for the meta-analysis.

| **Author and Publication Year** | **Type of BC**  Invasive or invasive and DCIS | **Density index** | **Highest density**  **category vs. lowest**  **density category** | **Numerical data**  *Lowest density category*  cases: non-cases  *Extremely dense* *breasts* cases:non-cases | **Crude OR**  **(95% CI)*** | **Maximally confounder-**  **Adjusted OR/HR/RR** and 95% CI** | **P value** |
| --- | --- | --- | --- | --- | --- | --- | --- |
| **Astley SM (2018)** | Both | VDG^1^ 5^th^ edition | VDG4 vs. VDG1 | VDG1 47:205  VDG2 189:570  VDG3 78:193  VDG4 24:46 | 2.92 (1.53-5.58) | 3.00^a^ (1.54-5.86) | Both statistically significant |
| **Brandt KR (2016)** | Both | VDG1 and Quantra BI-RADS^2^ | VDG 4 vs. VDG2  Quantra BI-RADS4 vs. Quantra BI-RADS2 | Volpara:  VDG 1 245:772  VDG 2 553:1274  VDG 3 677:1333  VDG 4 436:791  Quantra BI-RADS  QB1 134:482  QB2 919:2150  QB3 737:1368  QB4 121:170 | - | Volpara:  1.82^b^ (1.49-2.21)  Quantra:  1.94^b^ (1.48-2.54) | Both <0.0001 |
| **Engmann NJ (2019)** | Both | BI-RADS^2^ 5th edition | Not applicable | Premenopausal:  A 21:110  B 138:440  C 318:678  D 186:279  Postmenopausal:  A 229:853  B 716:1,686  C 563:1,118  D 119:198 | - | Only reported per BMI^3^ group and 1 unit increase. |  |
| **Eriksson M (2017)** | Both | STRATUS cBI-RADS^4^ | cB4 vs cB1 | cB1 26:193  cB2 174:829  cB3 189:593  cB4 44:117 | - | 4.9^c^ (2.8-8.6)  4.8^d^ (2.6-8.8) | Not reported |
| **Jeffers AM (2017)** | Invasive | BI-RADS^2^ and VDG^1^ | BI-RADS D vs. BI-RADS B  VDG 4 vs. VDG 2 | BI-RADS  A 10:53  B 46:112  C 57:87  D 12:22  VDG  VDG1 20:54  VDG2 19:54  VDG3 54:99  VDG4 32:67 | - | BI-RADS:  2.06^e^ (0.85-4.97)  VDG  2.05^e^ (0.90-4.64) | BI-RADS  <0.001  VDG 0.02 |
| **Kerlikowske K (2017)** | Invasive | BI-RADS^2^ | BI-RADS D vs. BI-RADS B | BI-RADS  A 233:731  B 668:1,557  C 600:1,115  D 219:283 | - | 2.01^f^ (1.61–2.51)  2.45^g^ (1.93–3.09) | Both statistically significant |
| **Puliti D (2018)** | Invasive and DCIS^5^ | VDG^1^ | Not applicable | All cancers  VDG1 27 out of 3,109  VDG2 48 out of 3,959  VDG3 73 out of 5,727  VDG4 68 out of 3,157  Invasive cancers  VDG1 26 out of 3,109  VDG2 35 out of 3,959  VDG3 49 out of 5,727  VDG4 56 out of 3,157 |  | Cases/person-years RR (95% CI)  VDG1–3 110/30390 3.6‰ reference  VDG 4 56/7513 7.5‰ 2.0 (1.5–2.8) | Not reported |
| **Román M (2021)** | Invasive and DCIS^5^  Invasive only | BI-RADS^2^ | D vs. B | All cancers  A 498:60,343  B 1,831;160,496  C 724:44,386  D 644;26,021 | - | No benign breast disease all cancers:  HR 2.27^h^ (2.07–2.49)  No benign breast disease invasive cancers only:  HR 2,59^h^ (2,31-2,90)  No benign breast disease, BI-RADS at latest screening examination HR 2,05^h^ (1.83-2.29) | Not reported |
| **Wanders JOP (2017)** | Invasive and DCIS^5^ | VDG^1^ | VDG4 vs. VDG1  VDG4 vs. VDG2 | All cancers  VDG1 58:10,458  VDG2 215:21,276  VDG3 189:15,856  VDG4 61:4,701 | - | VDG4 vs. VDG1  3.14^i^ (2.17 ; 4.55)  3.54^j^ (2.39 ; 5.24)  VDG4 vs. VDG2  1,76^i^ (1,32; 2,37) | <0.001 |

1. Volpara Density Grade (VDG); 2. Breast Imaging Reporting and Data System (BI-RADS); 3. Body Mass Index (BMI); 4. Computer-generated score categorizing breast density into four breast composition groups reflecting the Breast Imaging Reporting and Data System score (Stratus cBI-RADS); 5. Ductal Carcinoma in Situ (DCIS)

* Crude OR as calculated with the numerical data.; ** aOR = maximally confounder-adjusted odds ratio (OR), relative risk (RR), hazard ratio (HR), comparing breast cancer risk in the highest density category with that in the lowest density category as defined in that study, and their 95% confidence intervals (CI).

a. Adjusted for Tyrer-Cuzick score; and parity.; b. Adjusted for age and BMI.; c. Adjusted for age and BMI.; d. Adjusted for age; BMI; mammographic density; microcalcifications; masses; breast cancer in family; menopausal status and current use of HRT.; e. Age and race matched; adjusted for menopausal status, parity and BMI.; f. Adjusted for age, race/ethnicity, history of breast biopsy, family history of breast cancer.; g. Adjusted for age, race/ethnicity, history of breast biopsy, family history of breast cancer, BMI.; h. Adjusted for age at first screen, and year of screen.; i. Adjusted for age. Invasive cancers and DCIS included.; j. Adjusted for age. Invasive cancers only.

| **Study design** | **Age (years)** | **Number of cases/controls** | **Type of measurement** | **Partition*** | **OR**^1^ **(95% CI^2^)** | **Trend**** | **Adjustments** | **Ref^3^** |
| --- | --- | --- | --- | --- | --- | --- | --- | --- |
| Case-control | <50-70+ | 338/840 | VDG^4^ | VDG1 vs. VDG4 | Study 2:  3.00^a^ (1.54-5.86) | Yes | Tyrer-Cuzick score, parity | Astley |
| Retrospective case-control | 34-94 | 1,911/4,170 | VDG^4^ and Quantra BI-RADS^5^ | VDG 4 vs. VDG2  QB4 vs. QB2 | Volpara:  1.82^b^ (1.49-2.21)  QB:  1.94^b^ (1.48-2.54) | Yes | Age, BMI^6^ | Brandt |
| Nested case-control | <45 >65 | 2,290/5,362 | BI-RADS^7^ | NA^8^ | BI-RADS:  2.06 (0.85-4.97)  VDG^4^  2.05 (0.90-4.64) | - | None | Engmann |
| Prospective nested case-control | 40-79 | 433/1,732 | STRATUS cBI-RADS^9^ | cB4 vs. cB1 | 4.9^b^ (2.8-8.6)  4.8^c^ (2.6-8.8) | Unknown | Age; BMI^6^; mammographic density; microcalcifications; masses; breast cancer in family; menopausal status and current use of HRT^10^. | Eriksson |
| Case-control | <40 – 79 | 125/274 | BI-RADS^7^ and VDG^4^ | BI-RADS D vs. BI-RADS B  VDG 4 vs. VDG 2 | Only reported per BMI^6^ group and 1 unit increase. | Yes | Age, race, menopausal status, parity, BMI | Jeffers |
| Case-control | Mean (SD^11^) 59.5 yrs (12.1) | 1,720/3,686 | BI-RADS^7^ | BI-RADS D vs BI-RADS B | 2.0^d^ (1.61–2.51)  2.4^e^ (1.93–3.09) | Yes | Age, race/ethnicity, history of breast biopsy, family history of breast cancer, BMI^6^. | Kerlikowske |
| Cohort | 49-54 | 216 out of 15,952 | VDG^4^ | VDG4 vs VDG1-3 | Cases/person-years RR^12^ (95% CI)  VDG1–3 110/30390 3.6‰ reference  VDG 4 56/7513 7.5‰ 2.0 (1.5–2.8) | Unknown | None | Puliti |
| Cohort | 50-69 | 3,697 out of 294,943 | BI-RADS^7^ | D vs. B | No benign breast disease all cancers:  HR 2.27^f^ (2.07–2.49)  No benign breast disease invasive cancers only:  HR 2,59^f^ (2,31-2,90) | Unknown | Age at first screen, and year of screen, benign breast disease. | Román |
| Cohort | 50-75 | 523 out of 52,814 | VDG^4^ | VDG4 vs. VDG1  VDG4 vs. VDG2 | VDG4 vs. VDG1  3.14^g^ (2.17 ; 4.55)  3.54^h^ (2.39 ; 5.24)  VDG4 vs. VDG2  1,76^g^ (1,32; 2,37) | Yes | Age | Wanders |

Table F.2: Summary of the data from included articles for the meta-analysis.

*Categories of least and most widespread density from which odds ratios were calculated; ** Significantly increased risk of breast cancer across all categories of density analysed in study

a. Adjusted for Tyrer-Cuzick score; and parity.; b. Adjusted for age and BMI.; c. Adjusted for age; BMI; mammographic density; microcalcifications; masses; breast cancer in family; menopausal status and current use of HRT.; d. Adjusted for age, race/ethnicity, history of breast biopsy, family history of breast cancer.; e. Adjusted for age, race/ethnicity, history of breast biopsy, family history of breast cancer, BMI.; f. Adjusted for age at first screen, and year of screen; g. Adjusted for age. Invasive cancers and DCIS included.; h. Adjusted for age. Invasive cancers only.

1. Odds Ratio (OR); 2. Confidence Interval (CI); 3. Reference (Ref); 4. Volpara Density Grade (VDG); 5. Quantra Breast Imaging Reporting and Data System (QB); 6. Body Mass Index (BMI); 7. Breast Imaging Reporting and Data System (BI-RADS); 8. Not applicable (NA); 9. Computer-generated score categorizing breast density into four breast composition groups reflecting the Breast Imaging Reporting and Data System score (Stratus cBI-RADS); 10. Hormone replacement therapy (HRT); 11. Standard deviation (SD); 12. Relative Risk (RR)

# **Appendix G – Quality assessment**

Table G.1: Methodological quality assessment with the use of the Newcastle-Ottawa Scale.

| **Author and Publication Year** | **Astley SM (2018)** | **Brandt KR (2016)** | **Engmann NJ (2019)** | **Eriksson M (2017)** | **Jeffers AM (2017)** | **Kerlikowske K (2017)** | **Puliti D (2018)** | **Román M (2021)** | **Wanders JOP (2017)** |
| --- | --- | --- | --- | --- | --- | --- | --- | --- | --- |
| **Case control** |  |  |  |  |  |  |  |  |  |
| **Selection:** |  |  |  |  |  |  |  |  |  |
| Case definition adequate | Yes, through medical records* | Yes, through record linkage | Yes, through record linkage | Yes, through record linkage | Yes through record linkage | Yes, through record linkage | NA^a^ | NA^a^ | NA^a^ |
| Representativeness of cases | Representative* | Representative* | Representative* | Representative* | Not stated | Representative* | NA^a^ | NA^a^ | NA^a^ |
| Selection of controls | Community* | Community* | Community* | Community* | Community* | Community* | NA^a^ | NA^a^ | NA^a^ |
| Definition of controls | No mention of history of BC^b^ | No history of BC^b^ * | No mention of history of BC^b^ | No mention of history of BC^b^ | No history of BC^b^ * | No history of BC^b^ * | NA^a^ | NA^a^ | NA^a^ |
| **Comparability:** |  |  |  |  |  |  |  |  |  |
| Control for confounding factor(s): Age, BMI^c^, parity, menopausal status, etc. | >2** Tyrer-Cuzick score and parity | 2**  Age and BMI^c^ | 2*  Menopausal status and BMI^c^ | 8**  Age; BMI^c^; mammographic density; microcalcifications; masses; breast cancer in family; menopausal status and current use of HRT^d^. | 5**  Age; race; menopausal status; parity and BMI^c^. | 5**  Age, race/ethnicity, history of breast biopsy, family history of breast cancer, BMI^c^. | NA^a^ | NA^a^ | NA^a^ |
| **Exposure:** |  |  |  |  |  |  |  |  |  |
| Ascertainment of exposure (breast density) | Secure record* | Secure record* | No mention of blinding | Secure record* | Secure record, no blinding for BI-RADS^e^ | No mention of blinding | NA^a^ | NA^a^ | NA^a^ |
| Ascertainment of same cases/controls | Yes* | Yes* | Yes* | Yes* | Yes* | Yes* | NA^a^ | NA^a^ | NA^a^ |
| Non-response rate | Not stated | Not stated | Same rate for both groups* | Not stated | Not stated | Not stated | NA^a^ | NA^a^ | NA^a^ |
| **Cohort** |  |  |  |  |  |  |  |  |  |
| **Selection:** |  |  |  |  |  |  |  |  |  |
| Representativeness of cohort | NA^a^ | NA^a^ | NA^a^ | NA^a^ | NA^a^ | NA^a^ | Somewhat representative* | Somewhat representative* | Somewhat representative* |
| Selection of non-exposed cohort | NA^a^ | NA^a^ | NA^a^ | NA^a^ | NA | NA^a^ | From same cohort* | Frome same cohort* | From same cohort* |
| Ascertainment of exposure | NA^a^ | NA^a^ | NA^a^ | NA^a^ | NA^a^ | NA^a^ | Automated volumetric assessment* | No mention of blinding | Automated volumetric assessment* |
| Demonstration outcome not present at start | NA^a^ | NA^a^ | NA^a^ | NA^a^ | NA^a^ | NA^a^ | Yes* | No | No |
| **Comparability:** |  |  |  |  |  |  |  |  |  |
| Control for confounding factor(s): Age, BMI, parity, menopausal status, etc. | NA^a^ | NA^a^ | NA^a^ | NA^a^ | NA^a^ | NA^a^ | No | 2**  Age at first screen, and year of screen | 1 (age)* |
| **Outcome:** |  |  |  |  |  |  |  |  |  |
| Assessment of outcome | NA^a^ | NA^a^ | NA^a^ | NA^a^ | NA^a^ | NA^a^ | Record linkage* | Record linkage* | Record linkage* |
| Follow-up long enough for occurrence outcome | NA^a^ | NA^a^ | NA^a^ | NA^a^ | NA^a^ | NA^a^ | No (mean 28 months) | Yes* | Yes* |
| Adequacy of follow-up cohorts | NA^a^ | NA^a^ | NA^a^ | NA^a^ | NA^a^ | NA^a^ | No statement | >80% follow-up* | No statement |
| **Total score** | 7 | 7 | 5 | 6 | 5 | 6 | 5 | 7 | 6 |

a. Not applicable (NA); b. Breast cancer (BC); c. Body Mass Index (BMI); d. Hormone replacement therapy (HRT); e. Breast Imaging Reporting and Data System (BI-RADS)

# **Appendix H– Analyses**

**Forest plots**

*
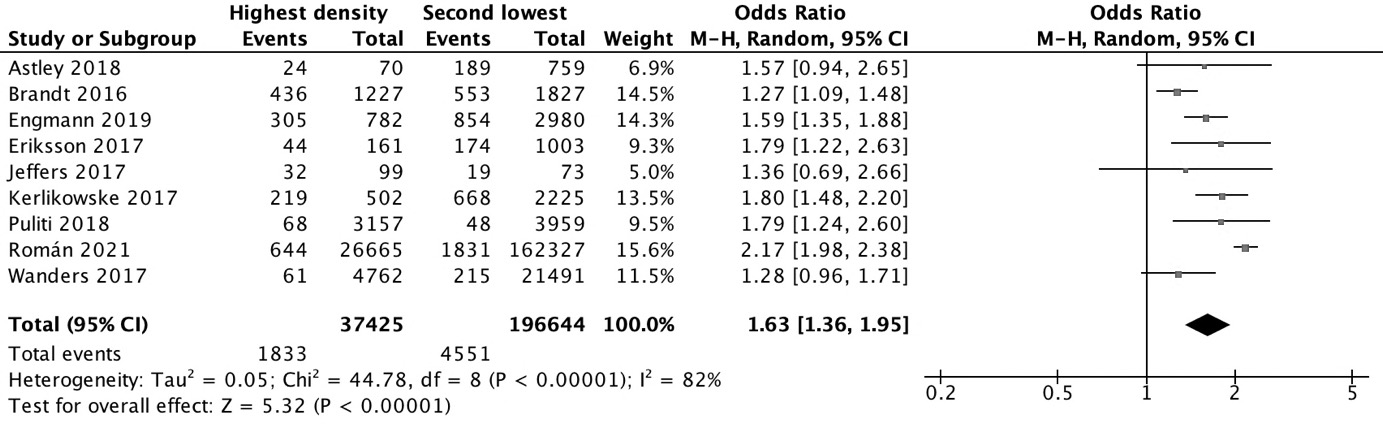
***Figure H.1:** Forest plot of the pooled risk (95% CI) for the nine included studies. Breast cancer risk for women with the highest breast density category (D) was compared to that of women with the second lowest density (B) category. Crude data were used. (Confidence Interval (CI))


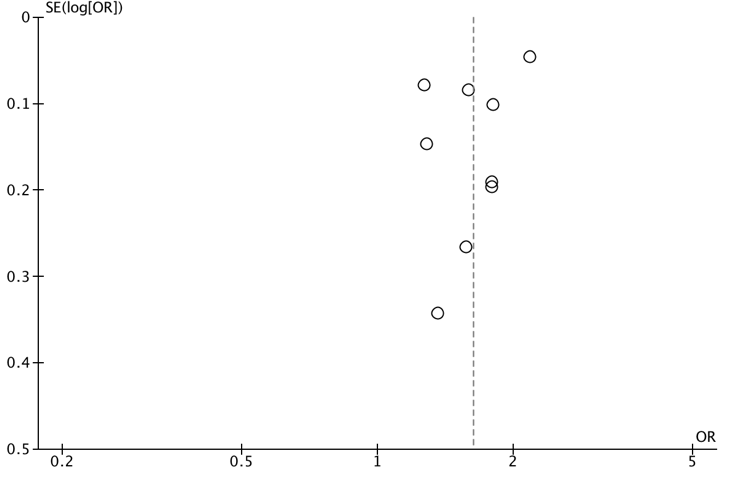


**Figure H.2:** Funnel plot corresponding to the forest plot of figure H.1. (Standard Error (SE); Odds Ratio (OR))

*
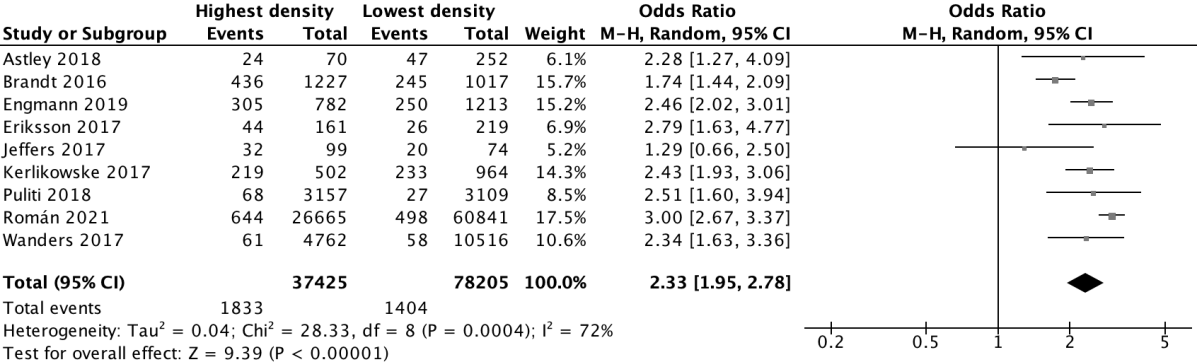
* **Figure H.3:** Forest plot of the pooled risk (95% CI) for the nine included studies. Breast cancer risk for women with the highest breast density category (D) was compared to that of women with the lowest density category (A). Crude data were used. (Confidence Interval (CI))


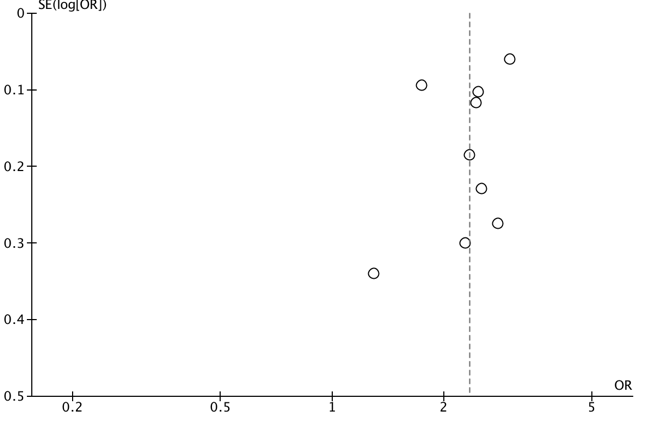

**Figure H.4:** Funnel plot corresponding to the forest plot of figure H.3. (Standard Error (SE); Odds Ratio (OR))


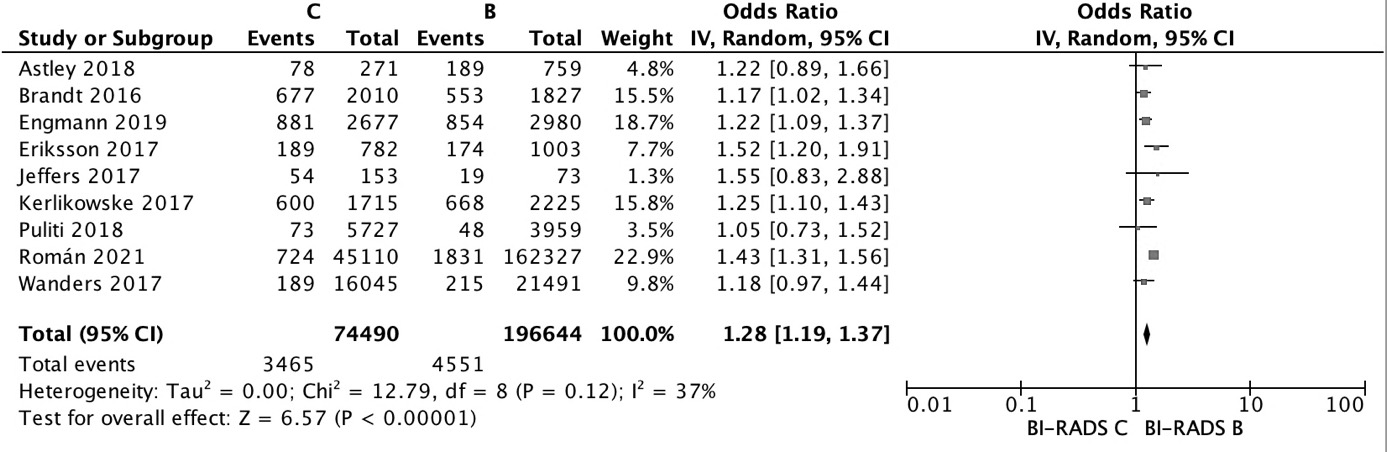


**Figure H.5:** Forest plot of the pooled risk (95% CI) for the nine included studies. Breast cancer risk for women with the second highest breast density category (C) was compared to that of women with the second lowest density category (B). Crude data were used. (Confidence Interval (CI))


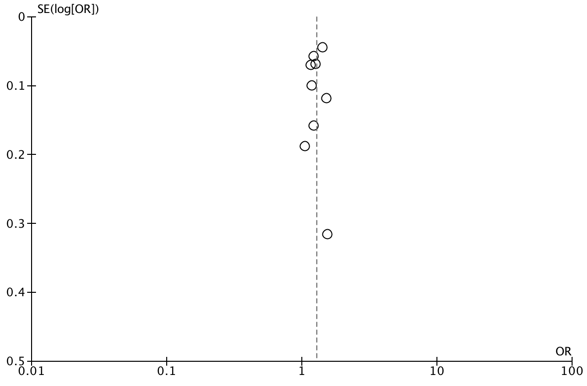


**Figure H.6:** Funnel plot corresponding to the forest plot of figure H.5. (Standard Error (SE); Odds Ratio (OR))


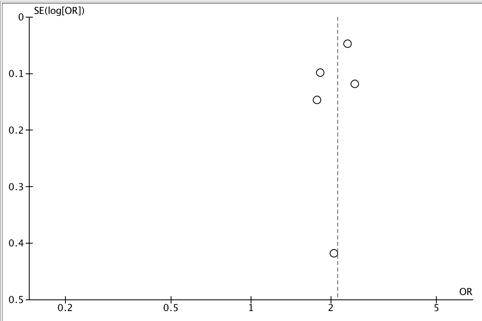


**Figure H.7:** Funnel plot corresponding to the forest plot of figure 2: of the pooled risk (95% CI) for five included studies. Breast cancer risk for women with the highest breast density category was compared to that of women with the second lowest density category. Maximally-adjusted data were used. (Standard Error (SE); Odds Ratio (OR); Confidence Interval (CI))

*
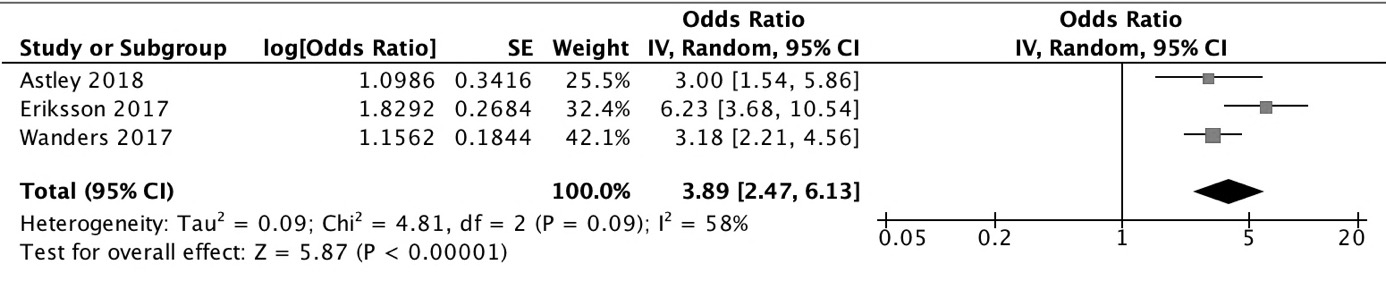
***Figure H.8:** Forest plot of the pooled risk (95% CI) for three included studies. Breast cancer risk for women with the highest breast density category was compared to that of women with the lowest density category. Maximally-adjusted data were used. (Standard Error (SE); Confidence Interval (CI))

*
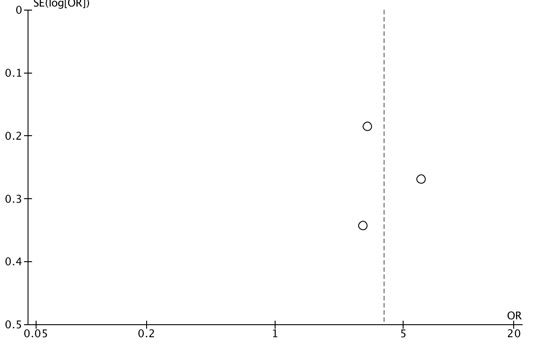
***Figure H.9:** Funnel plot corresponding to the forest plot of figure H.8. (Standard Error (SE); Odds Ratio (OR))

**Forest plots for stratified analyses and sensitivity analysis**

**
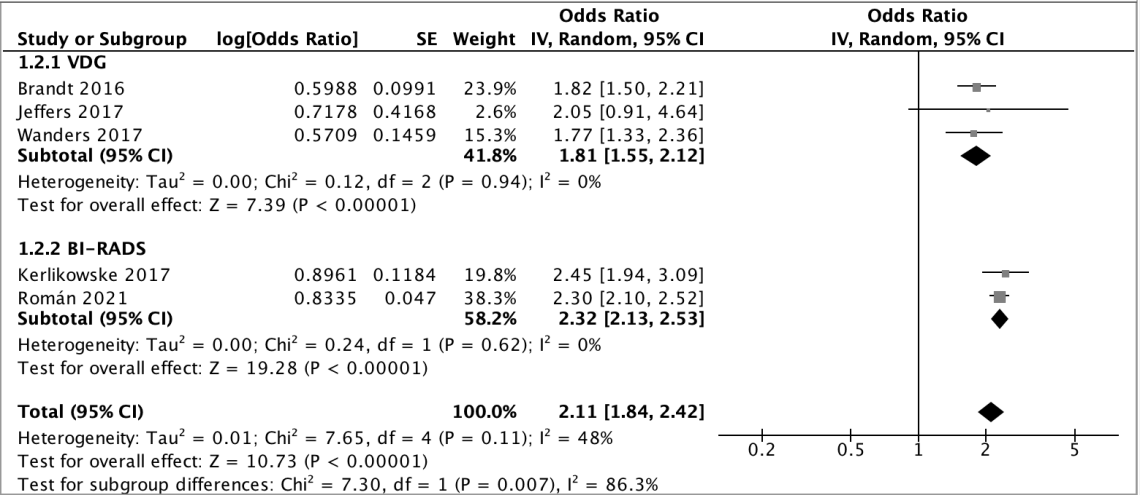

Figure H.10:** Forest plot for the stratified analysis per type of breast density index: Volpara density grade (VDG) vs. BI-RADS (Breast Imaging Reporting and Data System). The pooled risk (95% CI) for the five included studies were presented. Breast cancer risk for women with the highest breast density category was compared to that of women with the second lowest density category. Maximally-adjusted data were used. (Standard Error (SE); Odds Ratio (OR); Confidence Interval (CI))


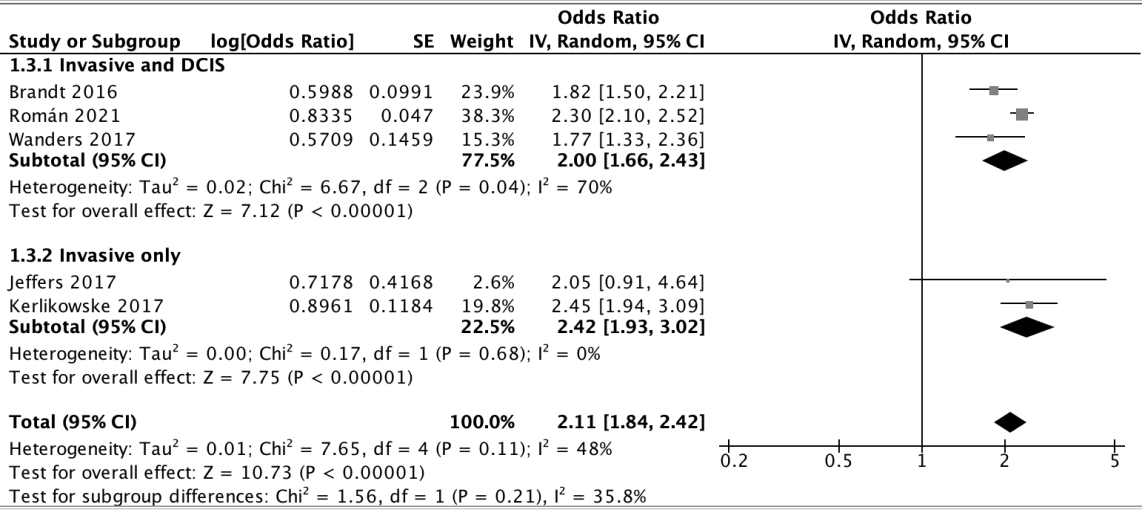

**Figure H.11:** Forest plot for the stratified analysis per type breast cancer outcome: invasive cancers and Ductal Carcinoma in Situ (DCIS) included in analyses vs. invasive cancers only. The pooled risk (95% CI) for the five included studies were presented. Breast cancer risk for women with the highest breast density category was compared to that of women with the second lowest density category. Maximally-adjusted data were used. (Standard Error (SE); Odds Ratio (OR); Confidence Interval (CI))

**
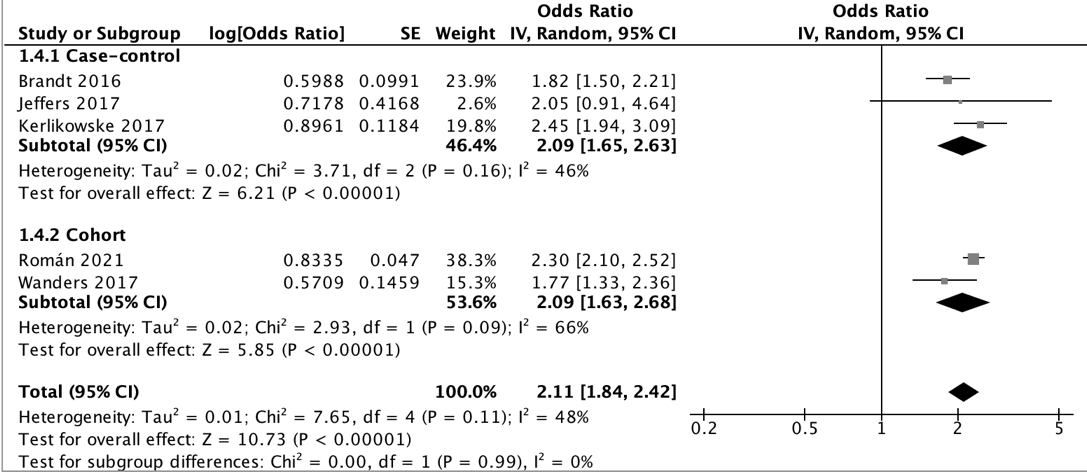

Figure H.12:** Forest plot for the stratified analysis per type of study design: case-control vs. cohort studies. The pooled risk (95% CI) for the five included studies were presented. Breast cancer risk for women with the highest breast density category was compared to that of women with the second lowest density category. Maximally-adjusted data were used. (Standard Error (SE); Odds Ratio (OR); Confidence Interval (CI))

*
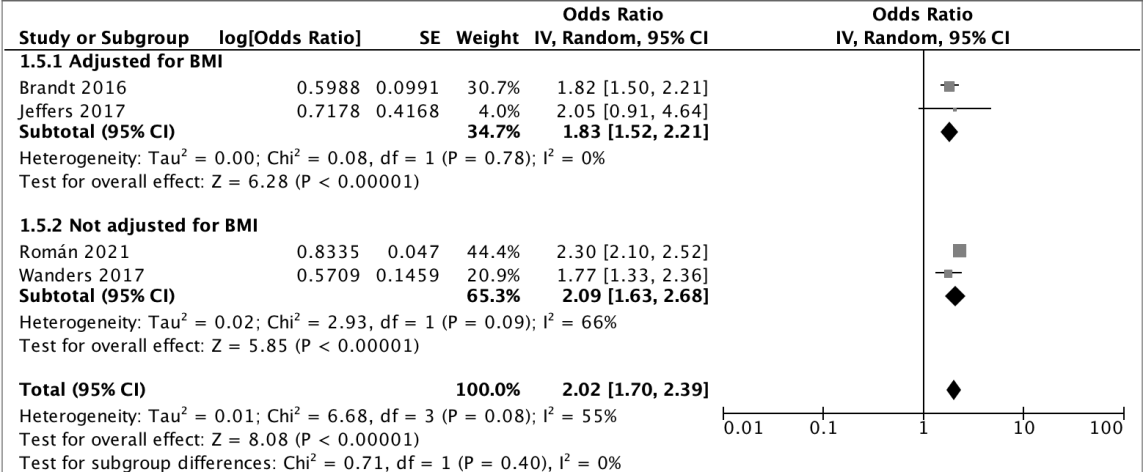
*

**Figure H.13** Forest plot for the sensitivity analysis: studies that had adjusted for Body Mass Index (BMI) vs. studies that had not adjusted for BMI. The pooled risk (95% CI) for the four included studies were presented (Kerlikowske et al. was excluded due to overlap in participants with Brandt et al.). Breast cancer risk for women with the highest breast density category was compared to that of women with the second lowest density category. Maximally-adjusted data were used. (Standard Error (SE); Odds Ratio (OR); Confidence Interval (CI))
